# Celebrating a body of work

**DOI:** 10.1371/journal.pcbi.1012441

**Published:** 2024-09-26

**Authors:** Jason A. Papin, Feilim Mac Gabhann, Virginia E. Pitzer

**Affiliations:** 1 Department of Biomedical Engineering, University of Virginia, Charlottesville, Virginia, United States of America; 2 Department of Biomedical Engineering, Johns Hopkins University, Baltimore, Maryland, United States of America; 3 Department of Epidemiology of Microbial Diseases, Yale School of Public Health, New Haven, Connecticut, United States of America

Thanks to a call-out from editorial board member Francis Ouellette, we recently realized that in September 2023, *PLOS Computational Biology* hit the milestone of publishing its 10,000th article [1]. This achievement happens as we approach our 20th anniversary as a journal [2]. The recognition of such milestones catalyzes an opportunity to reflect on the contributions we all have made to this ever-growing field.

We often celebrate singular achievements in science—a paper that is cited thousands of times [3], a software tool that is widely adopted by our field [4], or a prize that recognizes the achievement of an individual scientist. These celebrations highlight examples of impactful and field-changing contributions. However, recognizing only such singular contributions typically also means recognizing only a few members of a scientific community. Often under-appreciated is the fact that science, and computational biology specifically, is very much a community of practitioners [5] and that all members of the field benefit from the efforts of all that contribute to it. Together, we can achieve more progress than we ever could achieve alone.

Celebrated singular achievements benefit from a substantial body of work behind them. That paper cited thousands of times? It cites 49 papers itself. That software tool used widely by the field? That software tool is built on several previous iterations which involved many additional scientists. And both of the papers reporting those contributions required the scientific input from multiple editors and expert reviewers. A scientist celebrated for their singular achievement? In one notable speech [6], they acknowledged the work of many lab members and collaborators who were intimately involved in the celebrated discoveries, specifically acknowledging the importance of collective efforts in advancing science.

Let’s recognize wherever possible the staff, technicians, students, fellows, and principal investigators that work together to make the contributions that are celebrated. We love seeing scientific presentations where the lead investigator doing the talking calls out the members of their lab who made the specific contribution on a given slide, or perhaps even encourages the trainee to present the latest updates on the groundbreaking research. Let’s continue to innovate how we acknowledge scientific contributions through metrics that capture the diversity of impact beyond simply the number of citations an article garners [7]. *PLOS Computational Biology* continues to try to innovate how impact is measured with article-specific metrics [8], and to recognize the unsung efforts of the community through the publication of peer reviews [9], among other initiatives.

Just as scientific progress is built with and on the work of many, so too has our journal developed and flourished with the contributions of so many in our amazing field. From the authors of the 10,000 (and counting!) published papers, to the reviewers and editorial board members who volunteered their time to guide, assess, and improve them, to the journal staff who facilitated it all; over these past almost 20 years, too many people to count have made enduring contributions to our field. Whether your paper achieved broad impact and thousands of citations, or whether it had a more focused impact that provides key building blocks or the next key insight to unlock a path for others, we appreciate and salute the collective impact of all of us in bringing these 10,000 contributions of our field to the world.

Computational biology is a powerful force in advancing our understanding of the world. It is a “powerful play [that] goes on, and [we] may contribute a verse” [10]; let us celebrate our collective efforts to advance this field and the impact each of us has had.
